# Supplementary material for: Comparative genomics provides new insights into the diversity, physiology, and sexuality of the only industrially exploited tremellomycete: Phaffia rhodozyma
Source: BMC Genomics. 2016 Nov 9;17:901. doi: 10.1186/s12864-016-3244-7 (PMC5103461; doi:10.1186/s12864-016-3244-7)
Supplement: Additional file 6: — List of orphan genes with links to PFAM (related to Additional file 1: Table S1). (ZIP 1428 kb) [file 12864_2016_3244_MOESM6_ESM.zip › PFAM_UNIQUE/G04279_P.html]

#                                                                            --- full sequence --- -------------- this domain -------------   hmm coord   ali coord   env coord
  
# target name        accession   tlen query name           accession   qlen   E-value  score  bias   #  of  c-Evalue  i-Evalue  score  bias  from    to  from    to  from    to  acc description of target
  
#------------------- ---------- ----- -------------------- ---------- ----- --------- ------ ----- --- --- --------- --------- ------ ----- ----- ----- ----- ----- ----- ----- ---- ---------------------
  
G04279\_P             -            289 DUF605               PF04652.11   380       7.2    4.5   8.1   1   1      0.14       8.5    4.3   8.1   243   352   116   234    41   236 0.59 - Vta1 like  
G04279\_P             -            289 NABP                 PF07990.7    385       6.5    4.6   9.2   1   2    0.0045       6.7    4.5   4.7   142   201   110   166     6   176 0.70 - Nucleic acid binding protein NABP  
G04279\_P             -            289 NABP                 PF07990.7    385       6.5    4.6   9.2   2   2     0.013        20    3.0   0.2    15    72   174   237   157   247 0.72 - Nucleic acid binding protein NABP
